# Current diagnostics and treatment of fibrosarcoma –perspectives for future therapeutic targets and strategies

**DOI:** 10.18632/oncotarget.20136

**Published:** 2017-08-10

**Authors:** Daniela Augsburger, Peter J. Nelson, Thomas Kalinski, Andrej Udelnow, Thomas Knösel, Monika Hofstetter, Ji Wei Qin, Yan Wang, Arvid Sen Gupta, Susanne Bonifatius, Minglun Li, Christiane J. Bruns, Yue Zhao

**Affiliations:** ^1^ Department of General, Visceral und Vascular Surgery, Otto-von-Guericke University, Magdeburg, Germany; ^2^ Clinical Biochemistry Group, Medizinische Klinik und Poliklinik IV, University of Munich, Munich, Germany; ^3^ Department of Pathology, Otto-von-Guericke University, Magdeburg, Germany; ^4^ Institute of Pathology, Ludwig-Maximilians-University (LMU), Munich, Germany; ^5^ Department of Radiation Oncology, Ludwig-Maximilians-University (LMU), Munich, Germany; ^6^ Department of General, Visceral and Cancer Surgery, University Hospital of Cologne, Cologne, Germany; ^7^ Present address: Department of General, Visceral and Cancer Surgery, University Hospital of Cologne, Cologne, Germany

**Keywords:** fibrosarcoma, tumor microenvironment, therapeutic resistance, matrix metalloproteinases (MMPs)

## Abstract

Adult-type fibrosarcoma is a rare and highly aggressive subtype of soft tissue sarcomas. Due to the existence of other spindle-cell shaped sarcomas, its diagnosis is always one of exclusion. The likelihood of misdiagnoses between similar tumour entities is high, and often leads to inappropriate tumour treatment. We summarize here the main features of fibrosarcoma. When fibrosarcoma is appropriately diagnosed, the patient`s overall prognosis is generally quite poor. Fibrosarcoma is characterized by its low sensitivity towards radio- and chemotherapy as well as by its high rate of tumour recurrences. Thus it is important to identify new methods to improve treatment of this tumour entity. We discuss some promising new directions in fibrosarcoma research, specifically focusing on more effective targeting of the tumour microenvironment. Communication between tumour cells and their surrounding stromal tissue play a crucial role in cancer progression, invasion, metastasis and chemosensitivity. The therapeutic potential of targeting the tumour microenvironment is addressed.

## INTRODUCTION

Fibrosarcoma is a rare, highly malignant tumour of mesenchymal cell origin. It derives from pathologically transformed spindle shaped fibroblasts with an excessively high division rate. [1, 2] According to the WHO classification of soft tissue sarcomas, fibrosarcoma is defined as part of the fibroblastic/myofibroblastic sarcomas [3]. They are predominantly located either in deep soft tissue or adjacent to bones. Fibrosarcomas seldom derive from the cutis. Instead, it more frequently originates from tendons and fascias of the deep soft tissue. In addition, fibrosarcoma can occur inside bones, either as a primary or secondary tumor. Primary fibrosarcoma of bone may arise within the medullar canal. The periosteum can be a site of origin [4]. Pre-existing bone lesions, or bone damage induced by radiotherapy may give rise to the growth of secondary fibrosarcomas of the bone [5]. Two types of fibrosarcoma can be distinguished: the infantile/congenital-type fibrosarcoma, and the adult-type fibrosarcoma. In contrast to the infantile type, which the WHO has defined as an intermediate malignant rarely metastasizing tumour, fibrosarcoma occurring in adults is classified as a highly malignant tumour [3]. Fibrosarcoma was once thought to be the most common malignant soft-tissue sarcoma in adults [6]. According to current statistics from SEER, a program of the National Cancer Institute, fibrosarcomas occurring in adults account for 3.6% of all adult sarcomas [7]. Depending on the source, the patents sex may or may not play a role [3, 8]. Fibrosarcomas mainly arise in people between the ages of 25–79 [9]. The peak for the adult-type fibrosarcoma is between 30 and 60 years of age [8].

The diagnosis of fibrosarcoma is by one of exclusion [10]. Using immunhistochemical and molecular techniques, it is possible to further subdivide the various subtypes of fibrosarcoma which can be very similar in their morphology, tumour genetics and clinical manifestation. These include Low-Grade fibromyxoid sarcoma (LGFMS, Evans tumour), sclerosing epitheloid fibrosarcoma and myxofibrosarcoma. Moreover, other spindle-type tumours such as the monophasic fibrous synovial sarcoma, malignant peripheral nerve sheath tumour (MPNST), solitary fibrous tumour (SFT), aggressive fibromatosis as well as spindle-cell types of angiosarcoma, rhabdomyosarcoma, leiomyosarcoma and epitheloid sarcoma should be distinguished from fibrosarcoma [6, 5, 11, 8] Fibrosarcoma can occur near the skin surface. In these cases, spindle-cell malignant melanoma and sarcomatoid carcinoma should be excluded. The diagnosis of fibrosarcomas which arise secondarily in dermatofibrosarcoma protuberans (DFSP) is important, as they may respond to imatinib mesylate therapies [12–14]. Therefore, the diagnostic procedures should be thoroughly performed by all involved specialists. Anamnesis and a complete clinical examination should always precede imaging, histopathologic, immunhistochemic and moleculargenetic investigations. The best current therapy of fibrosarcomas is generous surgical removal [15].Even though the response rate of fibrosarcoma towards radio- and chemotherapy is very low, they are broadly used as a neoadjuvant and/or adjuvant tumour treatment. In this context, doxorubicin in combination with other chemotherapeutic agents is the major drug applied to patients.

To help ensure an accurate diagnosis and thus appropriate treatment plan, we summarize below the diagnostic steps and the typical anamnestic, clinical, imaging, histologic and immunohistochemical features of fibrosarcoma. The highly aggressive character of this tumor, its low response rate to chemotherapy, and the high rate of tumour recurrences contribute to its poor prognosis. The investigation of new treatment strategies is needed. We specifically discuss the potential importance of targeting the tumour microenvironment as a means of controlling tumor growth and chemosensitivity.

### Diagnosis of fibrosarcoma

### Anamnesis

A definitive cause of fibrosarcoma has not been identified. Nevertheless, genetic mutations[16] and some predisposing factors appear to influence the aetiology of fibrosarcoma [10]. Circumstances have been observed which may promote and constitute the tumour growth. These include scar tissues. for example in consequence of former burns, [17] and the insertion of foreign material e.g. vascular grafts and joint endoprotheses during surgery.[1, 18] Further predisposing factors include pre-irradiated tissue, a pre-existing dermatofibrosarcoma, well-differentiated liposarcoma, or a solitary fibrous tumour [3]. Anamnesis should always include detailed symptom-based questions of pain, paraesthesia, about changes in the size and consistency of the soft tissue mass, as well as about former lesions, surgical interventions and prior radiation therapies. [19] Anamnesis should always include a thorough medical and family history [20].

### Physical examination

Patients with unclear and potentially malignant soft tissue masses should be thoroughly examined for information about its location, size, shape, consistency and relationship to the surrounding tissue. Determination of the range of motion of nearby joints, a complete neurovascular examination as well as palpation of regional lymph nodes in view of tumour metastasis should be part of the physical examination [19, 20].

Fibrosarcoma mainly arises in regions consisting of collagen-rich connective tissue. Often, adult-type fibrosarcoma occurs in the lower extremities, especially in the area around the thighs and knees, the arms and the trunk, [9, 21]. In contrast, the diagnoses of fibrosarcoma of the retroperitoneum, mediastinum, head or neck is rarer. Fibrosarcomas are often found in deep soft tissue. The tumour mass is characterized by a firm consistency, a spherical shape, a sharp demarcation from the surrounding tissue, and an average size of 3–8 cm [8]. Due its sometimes deep localization, the rather unspecific and often painless soft-tissue swelling, this tumour may remain undetected for a long period of time (“tip-of-the-iceberg” phenomenon). [8, 10]. Symptoms arise when the surrounding tissue and/or organs are compressed by the infiltrating tumour. Depending on the tumour location micturition disorders, pain, disturbed blood circulation and movement restrictions may occur [9]. Final stages of fibrosarcoma may be accompanied by anorexia, weight loss and a reduced performance. Deeply localized, over 5cm in size, with pain associated with the mass, and steadily growing lumps are suspicious to malignancy. [15, 21], Patients should be referred to sarcomas reference centres for further imaging and biopsy and definitive diagnosis [22].

### Imaging diagnostics

If the anamnesis and physical examination suggest a potential soft tissue tumour, radiological imaging (Table 1) is the next step. Radiological imaging plays an important role in confirming a diagnosis, in assessing the tumour`s extent, in guiding biopsy and in determining the stage of disease [21] (Table 2). A multi-disciplinary evaluation of the images by oncologists, radiologists and pathologists is highly recommended. The procedure of choice in imaging soft tissue tumours of the extremities, the pelvis or the trunk, is the magnetic resonance imaging (MRI) [21, 22], accompanied by the application of contrast medium to assess vascularisation and necrosis. Soft structures including muscle, fat, nerves and vessels as well as necrotic, haemorrhagic and oedematous degenerations are additionally demonstrated by MRI. The tumour’s growth and size, its margin, the signal density, homogeneity and the distribution of contrast accumulation can be determined.

**Table 1:** Diagnostic imaging of fibrosacoma

**MRI**	**CT**	**X-ray**	**US**
*T1W-MRI* - inhomogeneous - hypo- to isointense *T2W-MRI* - inhomogeneous - hyperintense - contrast accumulation in tumour periphery + osteolysis, corticalis destructions, soft tissue indurations (bone involvement)	- homogeneous - weak signal amplification of CM	- denser than muscle - tumour calcifications (rarely) - bone may be eroded or saucerised with minimal periostal reaction	- heterogeneous - ill-defined margins

Diagnostic imaging of fibrosacoma [8, 10, 11, 21, 31].

Radiologic imaging is important to confirm suspicion of tumour, to define its local extent, to stage the disease, to assess changes after treatment, and to detect tumour recurrences. Depending on localization and involvement of bone, various imaging modalities can be applied. Tumours of the extremities, pelvis and trunk are visualized by MRI. CT and radiography are recommended in case of bone involvement. Ultrasound plays a limited role in the diagnostic of soft tissue sarcomas. Knowledge about the typical presentation of fibrosarcoma in each imaging modality helps differentiate this tumour entity from other soft tissue sarcomas.

CM (contrast medium), CT (computing tomography), MRI (magnetic resonance imaging), US (ultrasound), T1W-MRI (T1-weighed magnetic resonance imaging); T2W-MRI (T2-weighed magnetic resonance imaging.

**Table 2:** TNM Staging system for adult soft tissue sarcomas UICC/AJCC ^*^ (2010) [105]

Stage	Grade of differentiation (FNCLCC)	Primary tumour	Local lymph nodes	Distant metastasis
IA	G1, GX	T1a	N0	M0
		T1b	N0	M0
IB	G1, GX	T2a	N0	M0
		T2b	N0	M0
IIA	G2, G3	T1a	N0	M0
		T1b	N0	M0
IIB	G2	T2a	N0	M0
		T2b	N0	M0
III	G3	T2a	N0	M0
		T2b	N1	M0
	Any G	any T	N1	M0
IV	Any G	any T	Any N	M1

^a^the tumour is exclusively located above the superficial fascia without invading the fascia

^b^the tumour is exclusively located underneath the fascia/ the tumour is located superficially with invasion of or through the fascia/ the tumour is located both superficial yet beneath the fascia

^*^International Union against Cancer (UICC)/American Joint Committee on Cancer (AJCC)

TNM Staging system for adult soft tissue sarcomas UICC/AJCC ^*^ (2010), Histologic grade (G), GX: grade cannot be assessed; G1: low-grade; G2, G3: high-grade, Primary Tumour (T), TX: primary tumour cannot be assessed; T0: no evidence of primary tumour; T1: size > 5 cm; T1a: superficial tumoura; T1b: deep tumourb, T2 size < 5 cm; T2a: superficial tumoura ; T2b: deep tumourb, Regional lymph nodes (N), NX: regional lymph nodes cannot be assessed; N0: no regional lymph node metastasis; N1: regional lymph node metastasis, Distant metastasis (M), M0: no lymph node metastasis; M1: regional lymph node metastasis.

Alternatively, computed tomography (CT) or X-ray can help detect bone involvement. CT of the chest and abdomen/pelvis or an MRT of the whole body or positron emission tomography (PET)-CT can assist in the detection of distant metastases. CT is also recommended for retroperitoneal located tumours.[21, 22], More rarely ultrasound can be used to help distinguish between benign, cystic and malignant, rather than solid tumour formations, and should always be followed by MRI or CT [22].

In radiological imaging, fibrosarcomas appear as unspecific, often intramuscular localized, ovoid lesions. Its margins are slightly irregular.[11] A fibrosarcoma`s growth is characterized by displacing the surrounding tissue. Consequently, the impression of so-called pseudocapsules is created in the sectional view [21].

### Biopsy

There are different biopsy procedures for soft tissue sarcomas. It can be done by incisional or excisional biopsies conducted via open surgery. A minimal invasive procedure such as fine needle aspiration (FNA) biopsy or the core needle biopsy can be used [23–25]. The advantages of minimal invasive biopsies over open biopsies include a low rate of perioperative complications and reduced risk of tumour cell contamination. However, an accurate diagnosis can only be made if the tissue samples derive from distinct areas within the tumour, and if the material removed is sufficient for histological typing and grading. Due to the often insufficient amount of tissue obtained, FNA biopsies have been criticized and are often thought to be unsuitable as a diagnostic device. FNA biopsy in fibrosarcoma diagnostics is only recommended if the cytological finding, which should be interpreted by an experienced pathologist, is compared with prior clinical and imaging findings [26]. In Oncological Centres, for example, where interdisciplinary communication and expertise can be provided, the sensitivity of FNA is about 95% [27]. The sensitivity of core needle (tru-cut) biopsies is even higher [24]. While FNA biopsies play a limited role in the primary sarcoma diagnosis, it can be used to confirm tumour recurrences and nodal metastases. In contrast to FNA, the amount of tissue obtained by core-needle biopsies is generally sufficient which makes CT-guided core-needle biopsy a robust diagnostic procedure.[22] If the tumour exceeds the size of 3 cm and/or if minimal invasive methods have failed, surgical biopsies are indicated. Soft tissue tumours ranging between 3 and 5 cm in size should be biopsied via excisional biopsy. If the tumours exceed the size of 5cm, incisional biopsies can be conducted where only a part of the tumour is resected [19, 21]. The material obtained forms the basis for determination of the histological type and grade (Table 3) and subsequent immunohistochemical investigation.

**Table 3:** Histopathologic grading of fibrosarcoma

		Score 1	Score 2	Score 3
Score A Tumour differentiation score		Sarcomas closely resembling normal adult mesenchymal tissue Well differentiated fibrosarcoma	Sarcomas for which histological typing is certain: Classical fibrosarcoma	Embryonal and undifferentiated sarcomas: Poorly differentiated fibrosarcoma
Score B Mitotic activity score		0–9 mitoses per 10 HPF^*^	10–19 mitoses per 10 HPF	≥ 20 mitoses per 10 HPF
Score C Tumour necrosis score	no necrosis	≤ 50% necrosis	> 50% necrosis	
Final grade Grade1 (G1): Well differentiated, Low grade	Score A + score B + score C = 2 or 3			
Grade 2 (G2): Moderately differentiated, Intermediate grade	Score A + score B + score C = 4 or 5			
Grade 3 (G3): Poorly differentiated, High grade	Score A + score B + score C = 6,7 or 8			

A determination of the tumour`s malignancy is made by determining its degree of differentiation. The most widely recommended grading system is that of the French Federation of Cancer Centers Sarcoma Group (FNCLCC) [24, 98]. Three prognostically relevant factors, the tumour cell differentiation, mitotic index and the amount of necrosis, are scored independently. Finally, those scores are summed up and the grade of the tumour is assessed. The lower the combined score, the lower the grade, the less aggressive the tumour and the better a patient`s prognosis. There are four grades for sarcoma: GX (the grade cannot be evaluated), G1, G2 and G3. G1 tumours are considered low-grade. G2 and G3 are considered high-grade. [99] About 80% of fibrosarcomas are high-grade reflecting its overall aggressive character. [4, 30, 34].

^*^HPF (high-power field): 1 HPF = 0.1734 mm^2^.

### Histopathology of fibrosarcoma

**Figure 1:**
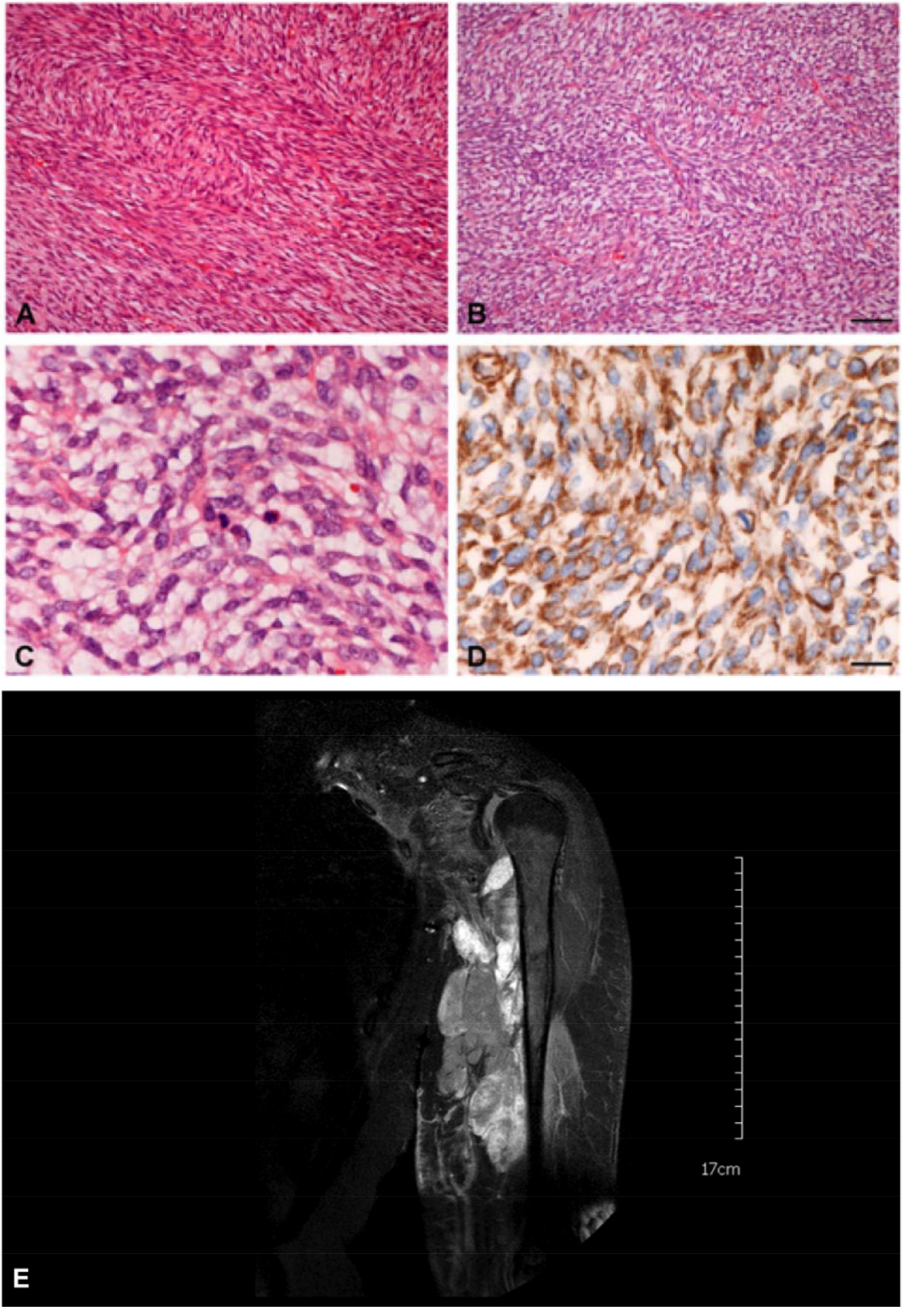
Histology and MRT of a typical case of an adult fibrosarcoma (FNCLCC grade 2) (**A**) Classical “herringbone” pattern and collagenous stroma. (**B**) More storiform area with lesser collagen content in the same tumour. Bar = 50 µm. (**C**) = Higher magnification showing mitotic activity. (**D**) = Positive Vimentin-immunohistochemistry. Other markers including EMA, pancytokeratins, α-SMA, desmin, S100, CD34 and CD117 were negative. Bar = 20 µm. (**E**) Magnetic Resonance Imaging of this patient shows a large hyper-intense tumor lesion in the left upper arm with infiltration und compression of the muscles and the brachial vessels and plexus.

Most soft tissue sarcomas can be assigned to one of the following five histomorphologic groups: pleomorphic pattern, epitheloid cell pattern, myxoid pattern, small round cell pattern and spindle cell pattern [28, 29]. Spindle cell sarcomas represent almost half of all sarcomas [30]. Fibrosarcoma belongs to the spindle cell type of soft tissue sarcomas. Spindle cells are characterized by its oval to fusiform nuclei, its uni- or bipolar cytoplasm and its lance shaped, tapered cells. The typical pattern of spindle-cell sarcomas derives from the fascicle-like cell arrangement. Fibrosarcoma is characterized by its parallely arranged monomorphic spindle-shaped fibroblasts. Often, these strands of fibroblasts are angled perpendicular to each other which causes the impression of a herringbone pattern (a case is shown in Figure 1) [2, 31, 3, 8] The nuclei are prominent with a variable number of nucleoli and an increased irregular, coarsed, granular chromatin; with limited cytoplasm [32]. Pleomorphism is rare. The amount of necrotic and hemorrhagic tissue, interstitial collagen and mitotic cells correlates with the stage of tumour malignancy. Malignancy also correlates with the degree of differentiation which is assessed by the FNCLCC grading system [24]. Depending on how much the malignant fibroblasts differ from normal tissue, fibrosarcoma can further be divided into well differentiated, conventional and poorly differentiated forms [23, 33]. The surrounding stromal tissue may take rather firm, keloid-like or loose or myxoid character [2].

### Tumour markers used in the diagnosis of fibrosarcoma

### Immunohistochemic (IHC) marker

Histopathology alone is not sufficient for a clear distinction between fibrosarcoma and other spindle-cell neoplasms. Immunohistochemistry (IHC) is applied in the diagnostics of fibrosarcoma where specific antibody reagents allow the detection of differential diagnostically important tumor markers [10, 6, 34–36], Tumour markers are molecules such as cell surface antigens, oncofetal antigens, enzymes, receptors, hormones, oncogenes or cytoplasmatic proteins. They are either overly expressed by the malignant cells themselves, or by the body’s reaction to the tumour.[37] They may be detected on the tumour cell surface, in the surrounding tumour microenvironment, in blood or urine. Tumour markers are used as part of therapy monitoring, process control and monitoring for tumour recurrence. The use of IHC markers is crucial for the differential diagnosis of fibrosarcoma [5, 8, 38–40].

The positive staining of vimentin demonstrates the mesenchymal origin of fibrosarcoma. Desmin, alpha smooth muscle actin (α-SMA) and muscle specific actin (MSA) belong to the most common myogenic markers. The S-100 protein is a neuronal marker that serves to exclude malignant peripheral nerve sheath tumors (MPNST). Positive CD 31, CD34 and factor VIII (von Willebrand factor) immunostains point to vascular malignancies e.g. spindle-cell angiosarcomas. Epithelial markers such as the epithelial membrane antigen (EMA), and cytokeratins can be helpful in the differential diagnosis of sarcomatoid carcinomas [35, 10]. In some mesenchymal tumours a differentiation of malignant cells into epithelial tissue can be observed during tumorigenesis. This phenomenon is called mesenchymal to epithelial transition (MET). Increased levels of E-cadherin and β-cadherin are associated with MET and are used to diagnose synovial sarcoma, epitheloid sarcoma and leiomyosarcoma, but not fibrosarcoma [41]. Cytokeratin expression can also be helpful in the differential diagnosis of synovial- and epitheloid sarcomas, which mostly expresscytokeratins 7, 8, 18 and 19 [36, 42]. Considered alone, immunohistochemical results are somewhat inconclusive as tumours usually express a number and variety of tumour cell- and tissue markers. Consequently, there is generally no individual immunohistochemical marker which is monospecific for a tumour type [36].

In fibrosarcoma, vimentin, is often the only positively stained marker [10, 34, 35, 3]. Sometimes muscle specific antigen (MSA) and/or smooth muscle actin (SMA) can be detected as a sign of myofibroblastic differentiation [10, 3]. In those fibrosarcomas which arise secondarily from either solitary fibrous tumour (SFT) or dermatofibrosarcoma, CD34 can sometimes be detected.

Ki-67, a cell cycle–associated nuclear antigen, is also used as a diagnostic marker for fibrosarcoma [2, 43, 44]. It is stained with anti-ki-67 antibodies such as MIB-1 which allow assessment of the so-called ki-67/MIB1 labelling index.[45]

### MicroRNAs- diagnostic markers

The lack of specifity of IHC markers has driven the search for additional biomarkers. In this context, microRNAs (miRNAs) have recently gained importance. Micro RNAs (miRNAs) are a class of small noncoding RNAs that bind to the 3′ untranslated regions (UTRs) of messenger RNA (mRNA), where they negatively regulate translation. It has been suggested that more than 60% of all mRNAs are controlled by miRNAs suggesting that they are major players in the posttranscriptional gene regulation.[46] Malignant cells aberrantly express specific miRNAs which may influence tumour proliferation, cell cycle control, apoptosis, differentiation and invasion. The miRNA expression profile appears to be cancer-type specific. To this end, (Table 4) it can also be used to distinguish fibrosarcoma from other spindle-cell sarcomas [47–49].

**Table 4:** MiRNA expression profile of HT1080

miRNA	miRNA expression profile of HT1080	miRNA targets	Target-dependent miRNA function
miRNA-373	↓ [106]	SIRT1^*^[107]	Onc
miRNA-409-3p	[108]	ANG mRNA^*^[109; 110]	TS
miRNA-520c	↑ [106]	mTOR^*^[107]	Onc
miRNA-181b-5p	↑↑ [106]	BCL2[111]	TS
miRNA-200b	↓ [112]	ZEB1, ZEB2 [113–115], SUZ12[116], EIF5A^*^[117]	TS
miRNA-200c	[108]	ZFHX1B^[118]^, ZEB1, ZEB2/SIP1^a^ ^[113–115]^, VEGFA[119], TIMP2[119], FBLN5[119], BMI1[120]	TS
miRNA-205	[108]	IL-24[121],ZEB1[113], ZEB2/SIP1[113], JAK2[122], VEGF-A[123; 124]	TS

The table shows human miRNAs and their expression profiles in the human fibrosarcoma cell line (HT1080). Depending on the target gene, miRNAs can either function as oncogenes or tumour suppressors. For each miRNA targets were picked out which play a role in the progression of various cancers. The tumour-dependent miRNA up- or downregulation determines whether a miRNA contributes to tumour progression or not.

^*^These targets, together with their corresponding miRNAs, have been investigated in fibrosarcoma (HT1080). SIP1 is a synonym symbol for ZEB2.

Onc (oncogene), TS (tumour suppressor) __ (expression not detectable), (↓ (decreased expression), ↑ (increased expression), ↑↑ (highly increased expression).

### Prognosis of fibrosarcoma

Fibrosarcomas and soft tissue sarcomas are prognostically evaluated by taking into account the age of the patient, the tumour size, depth and malignancy, the involvement of nerves, vessels, bone, the collagen density, as well as the metastatic potential and the formation of tumour recurrences [3, 8, 26, 21]. Prognostically unfavourable factors of fibrosarcoma include: (i) high histologic grade, (ii) large amount of tissue necrosis (> 50%), (iii) a high number of mitotic figures (> 20/10 hpf), (iv) a decrease of collagen fibres in favour of an increased cellularity, (v) deeply localized tumours, (vi) and tumours exceeding the 5 cm [8, 24]. Histopathological grading is considered to be the most important prognostic indicator, [26, 30]. High-grade fibrosarcoma patients with great risks for metastases will most likely benefit from adjuvant therapy [24]. 80% of adult-type fibrosarcomas are found to be high-grade malignancies [34]. Regardless of grade, the overall 5-year survival rate is about 40–60 % [8, 24, 34]. The ten year survival rate is 60% for low-grade, and 30% for high-grade tumours respectively [10]. Depending on the tumour grade, the patient`s age and the histology of the tumour margin, the recurrence rate lies between 12 and 79%, averaging in the 40% to 50% range need to be esbablished. In 10–20% of patients whose tumours had been adequately resected, recurrences occur within 5 years [8]. Haematologically spread metastases have been described in 9–63% of patients with adult-type fibrosarcoma [3]. In this context, the lungs and bones of the axial skeleton are the major site of metastatic spread. In a fewer number of cases, lymph node involvement is also seen [4, 3].

### Therapy

### Surgical intervention

Surgery represents the standard therapy of localized soft tissue sarcomas [22]. The surgical procedure depends on the tumour localization, its size and grade of malignancy [21]. In case of intramuscular localized soft tissue tumours, the affected muscle compartment should be resected en-bloc as part of the so-called compartment resection. In those cases, adjuvant radiation therapy is not indicated [22]. If on the other hand the tumours do not reach the muscle origin, and its insertion, or in case of an extracompartmental growth, a wide resection should be performed. If possible an R0, tumour margin-free, resection should be achieved in order to minimize the risk of local recurrences. This means that not only the tumour tissue itself, but also part of the adjacent healthy tissue has to be removed due to the infiltrative growth of fibrosarcoma. Even though a 2 cm margin is sometimes recommended, [21] a generally valid safety margin has not yet been determined. The reason lies in the patient-dependent involvement of anatomical critical structures such as nerves and vessels. In case of deep, high-grade, </> 5 cm large tumours, radiation therapy after an R0 resection is highly recommended. In case of other constellations of tumour grade, size and localization, the necessity of adjuvant radiation therapy in an R0 situation should be discussed in a multi-disciplinary fashion [22]. In case of R1/R2 situations, a reoperation should be performed if possible.

### Chemotherapy

In addition to surgery, radiotherapy and/or hyperthermia therapy, chemotherapy is a major category of treatment. By targeting and killing rapidly dividing and proliferating cells, such as malignant tumour cells, chemotherapeutical agents are broadly used to stabilize disease and for tumour remission. Adjuvant chemotherapeutic therapy in soft tissue sarcomas is very controversial, [22, 21] and is therefore not a standard treatment in these tumours. Patients with advanced cancers are the most likely to show some benefit. Generally, the number of poor/non-responders among fibrosarcoma patients is very high. This is in large part due to the pronounced drug resistance of the tumour. Human fibrosarcoma cells have been shown to establish co-resistances against vincristine, actinomcin D, vinblastine and etoposid when treated with the first-line chemotherapeutic agent doxorubicin.[50] This phenomenon of acquired multi-resistance is known under the term multidrug resistance (MDR).

Chemotherapy in patients with advanced stage fibrosarcomas is based on anthracyclines as the first-line treatment. In this context, doxorubicin is the most widely applied drug. Besides doxorubicin, response rates above 15% can be reached by actinomycin D and ifosfamide [51, 52]. A number of phase III studies have been conducted in order to assess the effect of adjuvant chemotherapy on the rate of local recurrences, on the rate of distant metastases, the disease-free-survival rate, and on the overall survival rate. An improvement of the overall survival has been detected in only 4–11% of sarcoma patients treated with chemotherapy.

In contrast to adjuvant chemotherapy, neoadjuvant treatment has been shown to be more effective. Patients with high-grade fibrosarcomas can benefit from a presurgical MAID (mesna, doxorubicin, ifosfamide, dacarbazine) treatment [53].

### Current therapeutic focus – the tumour microenvironment

As we have seen, the aggressive character, lack of treatment response to chemotherapy and the high rate of tumour recurrences contribute to the poor prognosis of patients with fibrosarcoma. Therefore, major efforts have been made to identify new ways to slow its proliferation and migration and to increase tumor sensitivity towards apoptosis inducing drugs such as the doxorubicin.

To this end, the tumour surrounding tissue, or stroma, has been the focus of new therapy approaches. The tumour microenvironment consists of two main components, the cellular component comprising tumor associated fibroblasts, smooth muscle cells, adipocytes, endothelial cells and immune cells. The second component is the extracellular matrix (ECM) which fills the intercellular space. The ECM consists mainly of proteoglycans, fibrous proteins, adhesion molecules and proteases, and is characterized by being high dynamic [54]. The interactions between the tumour cells and their microenvironment is important to cancer progression, invasion and metastasis. The following sections discuss fibrosarcoma microenvironments in which are thought to have a high therapeutic potential for the control tumour growth and the enhancement of chemosensitivity.

### Matrix metalloproteinases (MMPs) and their inhibitors

Matrix metalloproteinases (MMPs) [55, 56] are responsible for ECM reorganization and degradation which in turn are regulated by various inhibitors: (i) α2-macroglobulin, (ii) thrombospondin-1 and thrombospondin-2, (iii) membrane anchored glycoprotein RECK (reversion-inducing cysteine-rich protein with Kazal motifs) [57, 58] (i) tissue inhibitors of matrix metalloproteinases (TIMP-1, TIMP-2, TIMP-3, TIMP4), [59] (v) tissue-factor-pathway-inhibitor 2 (TFPI2),[60] (vi) procollagen C-terminal proteinase enhancer (CTPCPE) [61]. A prerequisite the tissue homeostasis is the balance between MMP activity and the presence of their specific inhibitors. Imbalances in the reorganization of the ECM in favour of tissue degradation, is physiologically found during embryogenesis and wound healing. Such imbalances are also seen in the tumour microenvironment. MMPs are produced by the tumour cells as well as the surrounding stromal cells [62]. The close interaction between the malignant cells and their microenvironment results in a paracrine activation of stromal cells, fibroblasts, adipocytes, smooth muscle cells as well as a paracrine attraction of immune cells, endothelial and mesenchymal progenitor cells by the tumour cells. Their activation also leads to the release of tissue degrading MMPs. Various factors have been shown to influence fibrosarcoma cells, these include: [63, 64] (i) the proinflammatory cytokines IL-6 and IL-8, (ii) fibroblast growth factor 2 (FGF2), (iii) macrophage inhibitory factor (MIF), (iv) osteopontin (OPN) and transforming growth factor beta (TGF- β). In general, the increased ECM degradation seen allows enhanced tumour invasion into surrounding tissue as well as increased metastasis by the removal of physical barriers. Tumour areas with invasive growth patterns are characterized by a high density of MMP expression. Additionally, ECM degradation results in the release of chemokines, TGF-β, proteins with RGD integrin binding sequences, and growth factors. Their subsequent activation of RAS-, MPK/ERK- and PI3K/Akt/mTor-signal pathways helps to drive tumour proliferation and motility. Many of these growth factors are also linked to increased angiogenesis which additionally has a positive effect on tumour growth [62].

So there is a general positive correlation between the amount of MMPs present and tumour progression. The higher the MMP concentration, the more advanced the cancer - the poorer the prognosis and overall survival. Controlling MMP activity within the tumour microenvironment is one way to locally control fibrosarcoma growth and metastasis.

### Specific inhibitors of MMPs

As a means to control ECM degradation and tumour progression, a series of broad spectrum MMPs inhibitors such as batimastat (BB-94), marimastat (BB-2516), GM 6001, CT1746, KB-R7787, prinomastat (AG3340), BMS275291, BAY 12-9566, CGS 27023A were developed. However, due to the high levels of side effects such as the musculoskeletal syndrome, most clinical phase III studies had to prematurely be curtailed [65, 66]. As an additional problem , therapeutic plasma levels often could not been reached. Thus to date, the synthetic broad spectrum inhibitors could not be shown to improve the survival rate. In fact, rather the opposite occurred. In some cases the unspecific inhibition of MMPs led to an acceleration of tumour progression which was thought to be due to the existence of “tumour-protective” MMPs. Their inhibition led to increased tumour growth. MMPs 3, 9, 11 and 19 possess both –tumour-progressive and protective characteristics. MMPs 8, 12 and 26 are largely protective proteases [67, 68]. The broad-spectrum inhibitors are also seen as less effective for the treatment of more advanced tumour diseases [65]. Due to the disappointing results of the broad spectrum inhibitors, the research has increasingly focused on the development of new inhibitors with a low side effect profile, and the exclusive inhibition of MMPs with tumour-progressive characteristics.

The MMPs expression profile is generally tumour-specific. Human fibrosarcoma cells express extracellular MMPs 1, 2, 3, 7 and 9 as well as the membrane type MMPs 14, 15, 16 [69, 63], MMPs 1, 2, 3, 9 and 14 in particular are thought to play a key role in tumor invasion, metastasis and angiogenesis [55]. N-Biphenylsulfonyl-*N*-Isopropoxy-Aminoacetohydroxamic (ARP 101) selectively inhibits MMP2 activity which is strongly increased in fibrosarcoma. *In vitro*, ARP 101 resulted in a decrease of tumour invasion [70]. Selective inhibitors of tumour type-specific MMPs may show less side effects but this remains to be seen.

### Intratumoural injection of TIMP-1-GPI

Because these enzymes play such central roles in tissue homeostasis, pronounced side effects are seen with systemically applied MMPs-inhibitors (MMPI) [65]. To help address this, a recent approach in sarcoma research is to locally increase MMPI concentration by injecting inhibitors directly into the tumour tissue. The normal surrounding tissue is largely uninfluenced and the systemic side effects are decreased. An intratumoral change in MMP activity status has been shown to lead to reduced primary tumour growth. In this context, TIMP-1, a broad spectrum MMPI with low toxicity has been evaluated .[71] Its systemic application showed disadvantages including low bioavailability, short half-life and the high amount of protein required for minimum effective doses (MED) [72]. A more recent approach is to increase the TIMP-1 concentration within the tumour tissue. One method to do this involves engineering the tumor tissue with expression vectors to enhance TIMP-1 production. [73, 74] Our group has developed a means of locally increasing the intratumoral TIMP-1 concentration by using a method called cell surface engineering. The principle involves the engineering of recombinant TIMP-1 protein to include a glycophosphatidylinositol-anchor (GPI-anchor). This lipid structure leads to efficient protein incorporation into cell membranes [75]. The particularly strong effect of TIMP-GPI-fusion proteins on tissue homeostasis and cell proliferation has been demonstrated in pathological wound healing specifically on hyperproliferating fibroblasts where an increase in apoptotic sensitivity and inhibition of cell proliferation was seen. [76, 77] TIMP-1-GPI was subsequently investigated as a therapeutic agent for the treatment of experimental fibrosarcoma [72]. Intratumoral injections of TIMP-1-GPI into fibrosarcoma-bearing mice led to a significant decrease in the tumour mass. The *in vitro* treatment of human fibrosarcoma cells with TIMP-1-GPI showed an inhibition of cell proliferation and migration as well as in an increased cell apoptosis and enhanced sensitivity to chemotherapy agents [72].

### Approaches to increase chemosensitivity

Fibrosarcoma shows pronounced resistance towards apoptosis inducing chemotherapeutic agents [52]. An increase in tumour chemosensitivity represents an important direction in fibrosarcoma research. There are several approaches that have been found to improve chemosensitivity, these include: (i) TIMP-1-GPI application, [72] (ii) suppressing potential chemoresistant cancer stem cells (CMCs) such as the side population (SP) cells e.g. via TIMP-1-GPI [72], and (iii) interrupting CAM-DR[78] by either homotrimeric collagen type I degradation or inhibiting the PI3K-Akt signalling pathway.

### Homotrimeric collagen type I – a promoter of CAM-DR

The reduced fibrosarcoma response towards therapeutic agents is linked in part to interactions between the tumour cells and their microenvironment [79]. This phenomenon, called environment mediated drug resistance (EMDR), describes a *de novo* development of drug resistance [80]. It is in contrast to acquired drug resistances where chemotherapy-induced genetic changes result in an increased extracellular transfer of drugs by enhanced expression of efflux pumps [81]. The EMDR is further subdivided into soluble factor mediated drug resistance (SFM-DR) or cell adhesion mediated drug resistance (CAM-DR) [78]. The autocrine and/or paracrine stimulation of tumour cells by growth factors, cytokines or chemokines leads to intracellular changes in chemotherapy-associated signal pathways and consequently decreases the therapeutic response rate. This is what is meant by the term SFM-DR. CAM-DR is mediated by adhesion between the tumour cells and components of the ECM. Integrins on the tumour cell surface moderate adhesion to ECM proteins such as collagens, fibronectins, laminins or stromal cell ligands. The activation integrin-mediated signal pathways, such as the PI3K-Akt pathway, results in enhanced drug resistance [82].

Collagen type I plays an important role in CAM-DR in fibrosarcomas. Two type I collagens have been found within the tumour tissue [83]. The heterotrimeric collagen type I is composed of two α1(I) and one α2(I)-chains. Homotrimeric isoforms are also expressed by tumour cells. These isoforms are largely resistant to MMP degredation [84]. In fibrosarcoma, homotrimeric collagen fibres can represent up to 50% of collagen type I present [83]. The autocrine interaction between homotrimeric collagen molecules and the tumour cells promotes CAM-DR and enhances tumour proliferation and migration, [85, 86]. The PI3K-akt signalling pathway is thought to play a central role in this biology [82]. Thus methods that help promote the degradation of the type I collagen isoforms may improve the efficiency of chemotherapeutic drugs. Because homotrimeric type I collagen is only seen in fetal and pathological tissue, its detection may help in the identification of residual tumour cells during surgery.

### The role of cancer stem cells (CSCs) in tumour initiation, proliferation and chemoresistancy

Recently the ‘cancer stem cell hypothesis’ has gained importance in cancer research [87, 88], It postulates the existence of a hierarchy within the tumour tissue where a small subpopulation of cells possess self-renewal, and stem cell-like characteristics. These cancer stem cells (CSC) are thought to be responsible for the initiation and regulation of tumour growth and have been called the roots of cancer [89]. CSCs are thought to have their own microenvironment referred to as the CSC niche [89]. CSCs are proposed to retain their ability to self-renew and to give rise to stem cell-derived cancer progenitor cells. Their environment helps protect the CSC from chemotherapeutic toxicity. Reports have suggested the existence of such a CSC niche in sarcomas [90]. CSCs are linked to the high recurrence rate of fibrosarcoma and its pronounced chemoresistancy and for this reason represent important targets for new treatment strategies for fibrosarcoma [91]. A specific fibrosarcoma stem cell marker has not yet been identified. In general CSCs are described to have the following characteristics: (i) they possess sphere formation ability, (ii) a high self-renewal potential, (iii) they behave invasively, (iv) they can be chemoresistant, (v) they possess tumour initiating potential, and (vi) they express the embryonic stem-cell related genes; Nanog, Oct3/4, Sox2, and Sox10. [92; 89] Hoechst staining is often used for the identification and isolation of a distinct subpopulation of CSC called side population (SP) cells [87]. SP are characterized by their expression of transmembrane efflux pumps which makes them highly resistant towards chemotherapy [93–95]. Some CSCs are also characterized by their upregulation of drug detoxifying enzymes such as the aldehyd dehydrogenases (ALDH) [87]. Fluorescent staining of ALDH allows the selection of ALDH positive cells which often possesses typical stem cell-like characteristics. Due to the fact that neither SP cells nor ALDH positive cells represent the total amount of CSC, it is necessary to combine both methods in addition to CSC markers for a more accurate characterization of the cells. The surface antigens CD24, CD90 and CD133 are thought to represent fibrosarcoma stem cell markers [92, 87, 96]. In the human fibrosarcoma cell line HT1080, 9% of cells were found to be ALDH-positive, 3.4–8.4% of the cells were CD133-positive, whereas only 0.3–0.54% belong to the SP, [92, 90, 96].

### Current clinical trials for soft-tissue sarcoma

The tumour microenvironment raises hopes for new sarcoma treatments [97]. This is confirmed by ongoing clinical trials. The therapeutic effect of olaratumab in combination with doxorubicin is currently being evaluated in a phase III clinical trial (ANNOUNCE) in patients with advanced or metastatic soft-tissue sarcoma [98]. Olaratumab is a platelet-derived growth factor (PDGF) receptor-alpha-blocking monoclonal antibody which blocks PDGF ligands from binding. Due to the positive results of the phase II trial, the combination of olaratumab and doxorubicin has been approved in the United States as a first-line therapy for patients with advanced soft-tissue sarcoma responding to anthracycline therapy. The trial completion is expected in 2020 [99–101].

The phase III placebo-controlled clinical trial of Anlotinib, a multi-target tyrosine kinase inhibitor, is another example of a clinical trial in progress to find a more effective treatment of advanced soft-tissue sarcoma by targeting the tumour microenvironment [102–104].

## CONCLUSIONS

The diagnosis of fibrosarcoma is one of exclusion, and an accurate diagnosis is a prerequisite for the design of an adequate treatment plan. Consequently, understanding the typical characteristics and features of this tumour type is important for accurate diagnosis. New markers such as miRNA expression profiles may represent an additional supportive diagnostic step in the identification of fibrosarcoma.

Due to its aggressive nature, the overall survival rate for a patient with adult-type fibrosarcoma is poor. The best prognosis is seen when the following points can be addressed: (i) complete surgical tumour resection with histological tumour-free margins (R0), (ii) the use of agents that lead to a reduction in tumour proliferation and migration and/or inducing remission via neoadjuvant or adjuvant therapy, (iii) preventing tumour invasion and metastasis.

A tumour`s proliferation and migration significantly correlates with the composition of the surrounding ECM components. The high concentration of MMPs within the tumour tissue results in a highly increased degradation of the ECM which consequently facilitates tumour growth as well as its spread to distant sites. In this context, intratumoral injections especially of the recently developed TIMP-1-GPI fusion protein [72] may represent a promising new treatment strategy. In addition, modulating the mechanisms which lead to CAM-DR may show benefit [78]. Homotrimeric isoforms of collagen type I [83] have been shown to play an important role in the acquirement of drug resistances.

**Figure 2:**
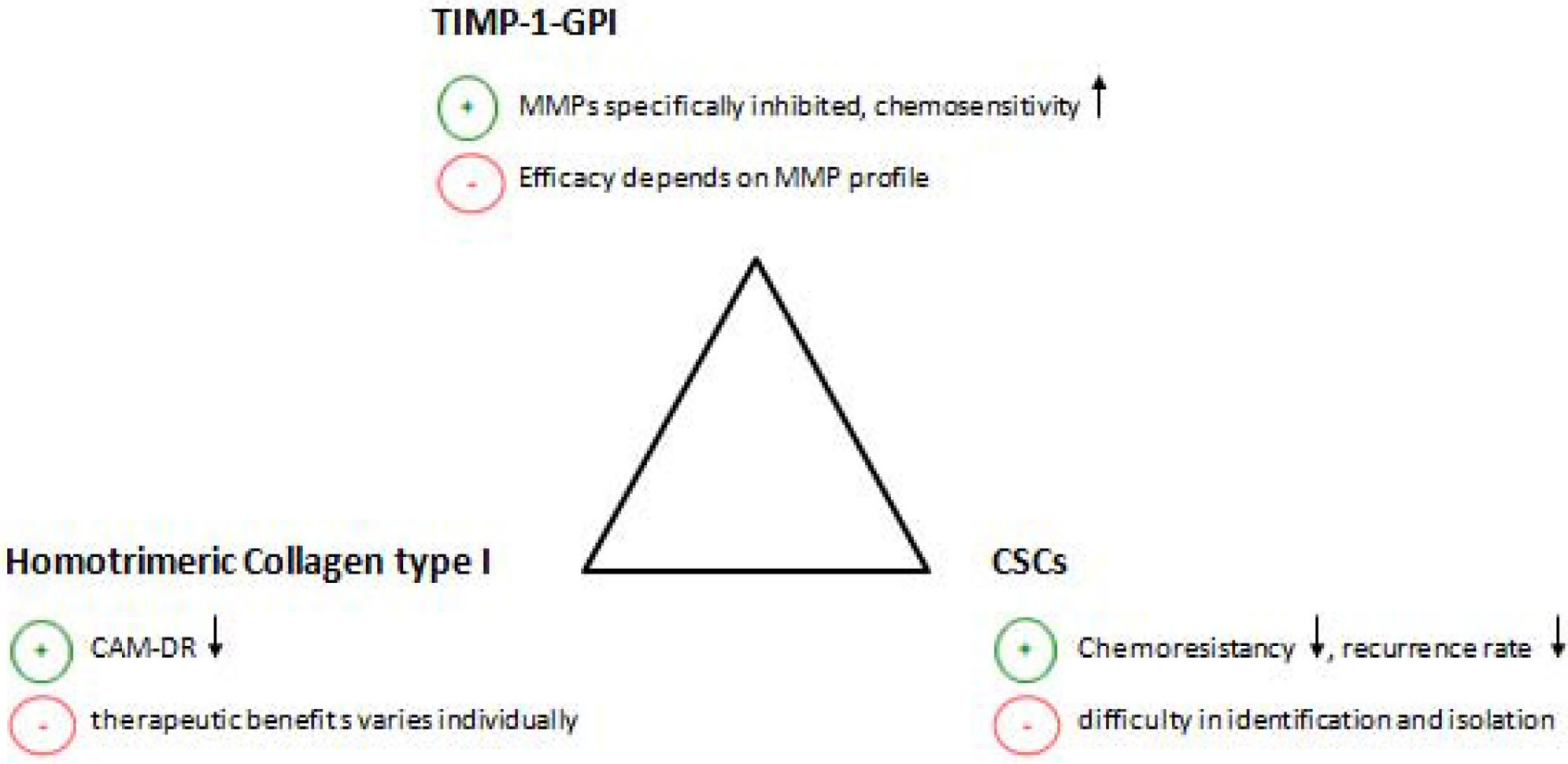
The figure shows three different approaches to improve the chemosensitivity of fibrosarcoma This includes the application of TIMP-1-GPI, the suppression of potential chemoresistant cancer stem cells (CMCs) and the interruption of CAM-DR by homotrimeric collagen type I degradation. The above figure further summarizes the pros (+) and cons (−) of each approach. CAM-DR (cell adhesion mediated drug resistance), CSCs (cancer stem cells), ↓ (reduction),↑(increase).

Similar to other treatment strategies the ones discussed above have their pros and cons (Figure 2). Hence the local application of TIMP-1-GPI inhibits specific MMPs and leads to a better chemosensitivity. However, the treatment efficacy is likely to depend on the MMP profile within the tumour tissue. Therefore, patients with fibrosarcoma may respond differently to TIMP-1-GPI treatment. The degradation of homotrimeric collagen I is an approach to decrease the cell adhesion mediated drug resistance (CAM-DR). However, the efficiency of this approach depends on the amount of homotrimeric collagen type I within the tumour tissue. Thus supposably not all the patients will benefit from this approach. Last but not least, the therapeutic potential of targeting the cancer stem cells lies in the decrease of chemoresistancy as well as in a lower recurrence rate. The challenge of this approach is to selectively identify and isolate those cells.

It should also be kept in mind that all the approaches mentioned in this article are pieces of the puzzle. The complexity of cancer is multidimensional. Therefore, the tumour microenvironment is only one dimension in the treatment of fibrosarcoma.

Targeting the tumour initiating cancer stem cells may be an important step in the treatment of fibrosarcoma. At present fibrosarcoma stem cell markers, even in combination, still fail to identify all CSC present in a sample. Further work in this field is urgently needed.
